# Muscarinic cholinergic receptors modulate inhibitory synaptic rhythms in hippocampus and neocortex

**DOI:** 10.3389/fnsyn.2014.00018

**Published:** 2014-09-05

**Authors:** Bradley E. Alger, Daniel A. Nagode, Ai-Hui Tang

**Affiliations:** ^1^Department of Physiology, University of Maryland School of MedicineBaltimore, MD, USA; ^2^Department of Psychiatry, University of Maryland School of MedicineBaltimore, MD, USA; ^3^Program in Neuroscience, Graduate School, University of Maryland BaltimoreBaltimore, MD, USA; ^4^Department of Biology, University of Maryland College ParkCollege Park, MD, USA

**Keywords:** optogenetics, GABA, interneuron, endocannabinoid, opioid, theta, cholecystokinin, parvalbumin

## Abstract

Activation of muscarinic acetylcholine (ACh) receptors (mAChRs) powerfully affects many neuronal properties as well as numerous cognitive behaviors. Small neuronal circuits constitute an intermediate level of organization between neurons and behaviors, and mAChRs affect interactions among cells that compose these circuits. Circuit activity is often assessed by extracellular recordings of the local field potentials (LFPs), which are analogous to *in vivo* EEGs, generated by coordinated neuronal interactions. Coherent forms of physiologically relevant circuit activity manifest themselves as rhythmic oscillations in the LFPs. Frequencies of rhythmic oscillations that are most closely associated with animal behavior are in the range of 4–80 Hz, which is subdivided into theta (4–14 Hz), beta (15–29 Hz) and gamma (30–80 Hz) bands. Activation of mAChRs triggers rhythmic oscillations in these bands in the hippocampus and neocortex. Inhibitory responses mediated by GABAergic interneurons constitute a prominent feature of these oscillations, and indeed, appear to be their major underlying factor in many cases. An important issue is which interneurons are involved in rhythm generation. Besides affecting cellular and network properties directly, mAChRs can cause the mobilization of endogenous cannabinoids (endocannabinoids, eCBs) that, by acting on the principal cannabinoid receptor of the brain, CB1R, regulate the release of certain neurotransmitters, including GABA. CB1Rs are heavily expressed on only a subset of interneurons and, at lower density, on glutamatergic neurons. *Exogenous* cannabinoids typically disrupt oscillations in the theta (θ) and gamma (γ) ranges, which probably contributes to the behavioral effects of these drugs. It is important to understand how neuronal circuit activity is affected by mAChR-driven eCBs, as this information will provide deeper insight into the actions of ACh itself, as well as into the effects of eCBs and exogenous cannabinoids in animal behavior. After covering some basic aspects of the mAChR system, this review will focus on recent findings concerning the mechanisms and circuitry that generate θ and γ rhythms in hippocampus and neocortex. The ability of optogenetic methods to probe the many roles of ACh in rhythm generation is highlighted.

## Introduction

The numerous effects that acetylcholine (ACh) has in the nervous system are mediated by both muscarinic (mAChR) and nicotinic (nAChR) receptors. Initially, attention focused on the mAChRs, following the classical experiments of Otto Loewi that showed that chemical transmission at synapses in the heart was mediated by ACh acting at mAChRs. With the recognition that nAChRs are also present in the brain and are directly relevant to the understanding of, e.g., the addictive potency of nicotine and its importance in schizophrenia, an enormous effort has gone into investigating the nAChRs in the central nervous system, although work continued on the molecular structure and pharmacology of the mAChRs. In addition, electrophysiological studies have provided a wealth of data on their cellular actions, the ion channels that they control, and their downstream biochemical mechanisms. Yet, despite these efforts, there remain important gaps in our knowledge of how the mAChRs affect neuronal circuits. Neuronal oscillations are among the most prominent and readily detected signs of neuronal circuit behavior. Certain oscillations, particularly those in the theta (θ, 4–14 Hz) and gamma (γ, 30–80 Hz) frequency ranges, are widely believed to be essential for the performance of various behavioral and cognitive functions. A general cholinergic agonist, carbachol (CCh), is often used to induce these rhythms which are mediated by mAChRs in hippocampus and neocortex in model experimental systems. However, a full understanding of the cellular and molecular mechanisms of rhythm generation has not been achieved. GABAergic inhibitory interneurons are key elements in rhythm generation, and mAChRs affect their behavior in many ways.

**Figure 1:**
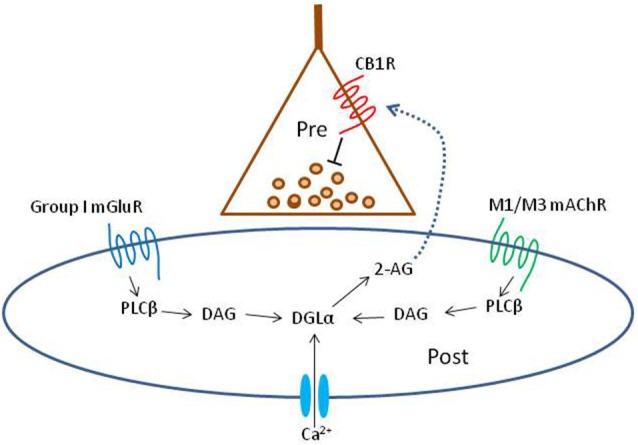
**Schematic summary diagram of the endocannabinoid system**. A presynaptic nerve terminal is shown synapsing on a postsynaptic cell. Agonist binding of either group I mGluRs or M1 or M3 mAChRs activates phospholipase Cβ (PLCβ) in the postsynaptic cell. The product of the reaction catalyzed by PLCβ, diacylglycerol (DAG), is metabolized by the enzyme diacylglycerol lipase α (DGLα) to form the endocannabinoid, 2-arachidonyl glycerol (2-AG). DGLα can also be activated by Ca^2+^ influx via a PLC-independent mechanism. 2-AG is membrane permeant and gains access through an unknown mechanism to the cannabinoid receptor, CB1R, on the presynaptic nerve terminal. Binding of CB1R by 2-AG inhibits transmitter release, mainly by inhibiting Ca^2+^ influx into the terminal, although other mechanisms can be involved. This is the simplest conventional model of the system and various details are controversial or have been omitted for the sake of simplicity; see a comprehensive review, e.g., Kano et al. (2009); for more information.

This review will highlight some new results on the generation of oscillations in hippocampus and neocortex; useful reviews of earlier work (e.g., Lawrence, 2008) have appeared. We will discuss the types of mAChRs, their influence on the some of the main interneuron subtypes and to lesser extent on principal cells. An emerging but still under-investigated theme is the ability of certain mAChRs to stimulate the synthesis and release of endogenous cannabinoids (endocannabinoids, eCBs), the natural ligands for the cannabinoid receptors (CB1Rs) in the brain (see diagram in Figure 1). In many regions, certain interneurons are heavily invested with CB1Rs while other interneurons have none; glutamatergic neurons often have far lower densities (up to 30 times lower) of the CB1Rs than do the interneurons. The great majority of CB1Rs are located on or near synaptic nerve terminals where their activation by exogenous cannabinoids and eCBs inhibit transmitter release. There has been little concerted effort to understand the implications of the mAChR-eCB link on complex nervous system activity, however. In view of the potency of mAChRs to mobilize eCBs, and thereby indirectly alter neuronal activity, it will be of great interest to work out the details and functional implications of this association. A key issue that has been difficult to explore with conventional methods concerns endogenously released ACh. The bulk of all experimental work on mAChRs and their physiological effects has been carried out either with gross tissue stimulation delivered by extracellular electrodes or perfusion with pharmacological agents. These methods lack specificity and selectivity of action, and the conclusions they permit are accordingly limited. We will discuss recent experiments in which optogenetic techniques have been used to probe the workings of the ACh system in unprecedented detail.

## Central cholinergic projection neurons associated with mAChR-induced rhythm generation

Almost all of the ACh in the hippocampus and neocortex comes from distal axons of cholinergic projection neurons that are highly concentrated in the basal forebrain (Lewis and Shute, 1967; see Woolf, 1991; van der Zee and Luiten, 1999; for review). This collection of nuclei includes the medial septum (MS) and lateral septum, the horizontal and vertical limb of the diagonal band of Broca (DBB), and the nucleus basalis magnocellularis (NBM). In rodents, cholinergic neurons in the MS/DBB project to olfactory-related structures, cingulate cortex, retrospenial cortex, medial prefrontal cortex, hippocampus and parahippocampus (Gaykema et al., 1989). The cholinergic projection from the MS/DBB to the hippocampus via the fornix/fimbria system is quite large, and selective ablation of the cholinergic cells in the MS/DBB or transection of the fimbria-fornix leads to a virtual loss of ACh fibers in the hippocampus (Lee et al., 1994; Naumann et al., 1994). Cholinergic neurons in the NBM project to the entire cortical mantle, with laminar projection patterns varying with cortical area. NBM also projects to the amygdala and olfactory bulb (see Woolf, 1991; van der Zee and Luiten, 1999 for reviews). An important anatomical feature of the ACh system in the brain is that cholinergic fibers only rarely (e.g., 3% in hippocampus and neocortex, Yamasaki et al., 2010) make classical morphologically defined synapses onto their target neurons (one-to-one, or “wired transmission”, Zoli et al., 1999). Rather, large vesicle-filled varicosities appear along the axons, and ACh is released into the local environment (Vizi and Kiss, 1998), where it diffuses in a paracrine-like way to receptors on target neurons and glia. This has been referred to as “volume conduction” (Zoli et al., 1999) and may be especially relevant for understanding mAChR-mediated effects, as they tend to have slow kinetics themselves and may involve the release of other modulators for which rapid kinetics is also not a key feature. A correlational EM study for the localization of M1 mAChRs and presynaptic synaptic specializations, including the presynaptic active-zone protein, bassoon, along cortical and hippocampal pyramidal cell (PC) dendrites, found that, unlike glutamate terminals and AMPA receptors, there was no close relationship between a cholinergic varicosity (identified by either the choline transporter, CHT1, or choline acetyl transferase, ChAT) near a dendrite and postsynaptic clusters of M1 receptors (Yamasaki et al., 2010). These findings appear to be consistent with the volume transmission mode. Sarter et al. (2009) question the relevance of the distinction between wired and volume transmission, since both can mediate responses with very slow kinetics, but admit that the difference may still be significant in the spatial domain—proximity of the release site to the receptors—which is the one most relevant to the present discussion.

## Distribution, action, and cellular localization of mAChR subtypes in hippocampus and neocortex

Muscarinic acetylcholine receptors (mAChRs) are G-protein coupled receptors of the Class A, rhodopsin-like family, with ACh being the main endogenous agonist (the ACh precursor, choline, reportedly induces γ activity in an atropine-sensitive way at 2–5 mM, Fischer et al., 2014). mAChRs are widely distributed throughout the central nervous system. The five subtypes, designated M1-5 (Bonner, 1989; Caulfield and Birdsall, 1998) can be divided into two broad groups based on their primary coupling to G-proteins. M1, M3 and M5 receptors (M1-class) are preferentially coupled to G_q/11_ proteins and activate phospholipase C, which initiates the IP3—diacylglycerol (DAG) cascade leading to intracellular Ca^2+^ mobilization, and activation of protein kinase C and mitogen-activated protein kinase (MAPK) pathways. M2 and M4 receptors (M2-class) couple to the pertussis-toxin sensitive G_i/o_ proteins and inhibit adenylyl cyclase activity.

Although widespread in the brain, there is considerable regional variability in the distribution of mAChR subtypes. Throughout the brain, M1 is the most abundant subtype and M5 the least. In the hippocampus and neocortex, M1 is present at high levels; M3 is present at moderate levels (though generally low elsewhere). M4 is very high almost everywhere in the brain, while M2 is found at much lower densities. M5 mRNA is relatively sparse except in hippocampal CA1 PCs and some scattered subcortical nuclei. M1-class receptors are often located on somato-dendritic regions of neurons, and their activation leads to membrane depolarization and increases in cellular excitability by enhancing the mixed-cation Na^+^/K^+^ current (*I*_h_), Ca^2+^ -dependent, non-selective cation current (e.g., Fisahn et al., 2002), and by inhibiting certain potassium channels, such as Kv7 (M-current), K_sAHP_, and K (“leak” channels) (e.g., Brown and Adams, 1980; Cole and Nicoll, 1983; Halliwell, 1990; Cobb and Davies, 2005; Lawrence et al., 2006b; Broicher et al., 2008). M2-class receptors frequently reside on presynaptic axonal terminals (although there are exceptions), and agonist binding can activate Kir3 potassium channels and inhibit some voltage-gated Ca^2+^ channels (especially Ca_v2.2_), which in turn hyperpolarizes the neuron or inhibits transmitter release (Hájos et al., 1998; Brown, 2010) (It should be noted that cholinergic axon terminals, including the axons of the MS/DBB fibers, generally express mAChRs, often M2-types, that probably act as presynaptic autoreceptors and regulate ACh outflow; this review will not cover this topic and interested readers can consult, e.g., Vizi and Kiss, 1998; Zoli et al., 1999 for reviews). While the exact cellular location and functional role of each subtype has not been fully elucidated, some correlations between different forms of cholinergic neuromodulation and the neurochemical identities of distinct neuron classes have been established in both hippocampus and neocortex.

## mAChRs on pyramidal cells and interneurons in hippocampus and neocortex

In the hippocampal CA1 region, PCs provide excitatory output to other cortical and subcortical areas, and carry information about spatial location and episodic memories (e.g., Eichenbaum, 2013). The functions of PCs are supported by local inhibitory circuits comprising more than 20 types of GABAergic interneurons (Freund and Katona, 2007; Klausberger and Somogyi, 2008; Whittington et al., 2011). The majority of these interneurons are morphologically and neurochemically distinct (Klausberger and Somogyi, 2008), yet their detailed functions have not been worked out. One broad distinction is based on whether the interneurons participate in feedback or feedforward inhibition; another is whether they synapse on dendritic or somatic regions of their target cells. There is some overlap between these classifications, with feedforward inhibition often mediated by dendritic targeting interneurons, and feedback inhibition mediated by perisomatic (i.e., including the soma and proximal dendrites 50–100 μm away) regions. However, there are many exceptions to this generalization (Bartos et al., 2011) and we will focus on the dendritic vs. perisomatic targeting distinction, which seems to be quite general across many brain regions.

Perisomatic targeting interneurons include two non-overlapping classes of basket cells (BCs): the parv- albumin-expressing (PV+), fast-spiking interneurons, and cholecystokinin-expressing (CCK+) regular-spiking interneurons (Freund and Katona, 2007; Bartos and Elgueta, 2012). A third type, the PV+ axo-axonic interneurons (often referred to as “chandelier cells” especially in the neocortex, e.g., Povysheva et al., 2013), innervates only the initial segments of PC axons; we will not discuss axo-axonic cells in detail.

The CCK+ and PV+ BCs differ in a number of fundamental features (Freund and Katona, 2007; Bartos and Elgueta, 2012). In addition to differences in firing patterns—non-accommodating, γ-synchronized action potentials in PV+ BCs; accommodating, poorly γ-synchronized action potentials in CCK+ BCs—one other distinction is that CCK+ BCs express the main receptor for the cannabinoids, CB1R, while PV+ BCs express the mu-opioid (μOR) receptor (Drake and Milner, 2002), which responds to certain opioids. Traditional anatomical and physiological evidence suggests that these two receptor populations have virtually no overlap (recent evidence that suggests this conclusion should be modified will be discussed below). These distinctions between PV+ and CCK+ BCs hold for both hippocampus and cortex, but not everywhere. For example, in the striatum PV+ BCs express CB1Rs (Kano et al., 2009). Additional differences between PV+ and CCK+ BCs can be found in their complement of mAChRs, as discussed below.

A well-studied representative of the dendritic targeting class of interneurons is the somatostatin (SOM)- and mGluR1a-expressing interneuron. Hippocampal interneurons in this class have their somata in the stratum oriens and the great bulk of their axonal arbor in the stratum lacunosum-moleculare, and are referred to as oriens-lacunosum moleculare (O-LM) cells. O-LM cells target the dendrites rather than perisomatic regions of PCs and have different phase preferences for firing within θ rhythm oscillations than do the BCs (or axo-axonic cells). Therefore O-LM cells play distinctive functional roles in regulating both PC excitability and temporal patterning of PC activity (Buzsáki, 2002; Klausberger and Somogyi, 2008; Bartos et al., 2011).

These classes of interneurons are not unique to the CA1 region; most other regions of the hippocampus and the neocortex have similar inhibitory configurations (Lund and Lewis, 1993; Curley and Lewis, 2012). The BCs in the neocortex have essentially the same properties as in the hippocampus, as do the axo-axonic cells (Curley and Lewis, 2012), although it appears the cortical axo-axonic cells have not received as much attention as the hippocampal ones have. In the cortex, the Martinotti cells are also SOM+ /mGluR1α+, and target PC dendrites, and seem generally analogous to the O-LM cells of the hippocampus. Therefore, understanding how mAChR activation regulates these interneurons and consequently the dynamics of hippocampal network activity may be applicable to the neocortex.

mAChRs are widely distributed on the principal cells of the hippocampus and neocortex. PCs in CA1-CA3 and granule cells in dentate gyrus all have abundant postsynaptic expression of M1 receptors, and weaker expression of M3 receptors (Levey et al., 1995). Activation of M1 or M3 usually increases cellular excitability. Therefore, the most dramatic direct effect of either exogenously applied cholinergic agonists or endogenously released ACh on PCs is a pronounced membrane potential depolarization and decrease in membrane conductance (Dodd et al., 1981; Cole and Nicoll, 1983; Pitler and Alger, 1990). This response, together with a decrease of the afterhyperpolarization (AHP; Cole and Nicoll, 1983) and the activation of a persistent, voltage-dependent sodium current (Yamada-Hanff and Bean, 2013), often results in sustained action potential firing (Cobb and Davies, 2005), particularly in hippocampal CA3, where the PCs form a strong recurrent intercollateral network. Neocortical PCs are similarly affected by muscarinic agonists (McCormick and Prince, 1986; Haj-Dahmane and Andrade, 1999).

For the inhibitory interneurons, the major muscarinic response is also depolarization, but with a less prominent associated change in cell input resistance. Generalizations are somewhat difficult to make however, given the diversity of mAChR subtypes, interneurons, and the specific distributions of mAChRs along the cells. For example, M1 is predominantly expressed on PCs but found in very low abundance, if at all, on GAD67-expressing interneurons, including O-LM cells (Yamasaki et al., 2010). These factors make the muscarinic modulation of interneurons much more complicated than that of the PCs. Besides the depolarizing effects of mAChR activation, some interneurons exhibit pure hyperpolarizations or biphasic responses, in which an initial hyperpolarization is followed by a secondary depolarizing phase (McQuiston and Madison, 1999a; Widmer et al., 2006; Bell et al., 2013); the hyperpolarizing responses are attributable to activation of M4 receptors, which activate inwardly rectifying K^+^ channels (Bell et al., 2013).

Both CCK+ and PV+ BCs are depolarized by mAChR activation, but some CCK+ Schaffer collateral-associated (SCA) cells also express M2 and M4 receptors to some extent and show biphasic responses when a cholinergic agonist is applied (Cea-del Rio et al., 2010, 2011). CCK+ BCs and SCA cells have strong expression of both M1 and M3 mAChRs, while PV+ BCs and axo-axonic cells express only M1 receptors in their somato-dendritic regions (Cea-del Rio et al., 2010, 2011). Therefore, CCK+ cells are more sensitive to ACh stimulation. More importantly, M3 receptor activation controls the mAChR-mediated increase in firing frequency, and both M1 and M3 mAChR activation is required for the full conversion of the spike AHP into a spike afterdepolarization. mAChR activation increases action potential duration and frequency and reduces spike adaptation in CCK+ cells, as in O-LM cells (Lawrence et al., 2006a), but not in PV+ cells (Cea-del Rio et al., 2010). On the other hand, the outputs of both types of interneurons are also modulated by mAChRs. PV+ cells express M2 receptors on their presynaptic axon terminals; activation of these receptors directly inhibits Ca^2+^ channels and suppresses GABA release (Hájos et al., 1998; Fukudome et al., 2004). ACh and muscarinic agonists also inhibit GABA release from CCK+ cells, but rather than directly activating presynaptic mAChRs, postsynaptic mAChRs on PCs reduce GABA release via an indirect retrograde signaling mechanism, as discussed in the section on eCBs and mAChRs, below.

The O-LM cells express both M1 and M3 receptors (Lawrence et al., 2006a) and generate large depolarizing responses upon mAChR activation (Kawaguchi, 1997; Widmer et al., 2006). Besides direct depolarization by inhibition of M-current, M1 or M3 activation also greatly accelerates action potential firing rate and generates a prominent suprathreshold afterdepolarization in these cells (Lawrence et al., 2006a,b).

## ACh generation and modulation of oscillations

### *In vivo* oscillations in hippocampus and neocortex

Rhythmic fluctuations in cell membrane potentials produce field potential oscillations. Depending on how many cells are synchronously involved, the oscillations can coordinate neuronal activity both locally and across brain regions and are considered to be essential for various cognitive functions. The two most prominent oscillations in the hippocampus are in the θ and γ ranges, which are often concatenated such that γ activity is observed “riding” on a θ carrier wave. ACh can have either a causal or modulatory role in these oscillations, most notably in the θ band, and the mechanisms by which ACh influences them are controversial. It is generally agreed that mAChRs play a more prominent role than nAChRs in rhythm generation.

θ-frequency firing is a basic operational mode of the hippocampus, and is proposed to underlie the formation of episodic memories and spatial maps of the environment (Buzsáki, 2005). θ can be detected in all layers of the CA1 hippocampus, although its amplitude and phase change with depth, with a current source located in s. pyramidale and a current sink near the border of s. radiatum and s.l.m. θ rhythms can modulate plasticity, particularly at the CA3-CA1 Schaffer collateral pathway. For instance, LTP is optimally induced if a train of electrical stimuli coincides with the peak of the θ rhythm, and stimulation given at θ frequency (“theta burst”) is optimal for the induction of LTP in CA1 neurons (Larson and Lynch, 1986).

Output from the MS/DBB is necessary for generating hippocampal θ-frequency rhythms *in vivo*, and lesioning the septum abolishes these rhythms and decreases the rate of learning by rats on a spatial maze task (Winson, 1978). However, the mechanisms of θ generation are not homogeneous, and differ depending on the behavior or state of an animal. “Type 1” θ occurs during active, exploratory behavior and is relatively insensitive to the mAChR antagonist atropine. This does not mean, however, that ACh has no role in Type I θ or associated behaviors. The mAChR antagonist, scopolamine, reduces the positive correlation between hippocampal θ and maze-running speed, and also diminishes the normally sharp spatial tuning of “grid” cells in the entorhinal cortex that provide a coordinate system for spatial navigation and memory formation (Newman et al., 2014). It is not known if scopalamine’s effects can be attributed to disruptions of the network oscillations, although this seems likely. In contrast to Type I θ, “Type II” θ occurs under urethane anesthesia and during REM sleep, and is abolished by atropine or selective immunolesioning of the septal cholinergic neurons (Stewart and Fox, 1989). Type II is often referred to as “atropine sensitive” θ. Injection of CCh, physostigmine, or muscarine into the hippocampus of an awake cat elicits θ rhythms in the EEG that can also be blocked by atropine, but not by the broad spectrum nAChR antagonist, mecamylamine (Konopacki and Goebiewski, 1992), again suggesting that nicotinic signaling does not play a major role in ACh associated rhythms. The causal role of ACh in Type II θ is not without controversy; ACh release appears to lag behind Type II θ onset during urethane anesthesia (Zhang et al., 2010). MS/DBB cholinergic neurons fire in a manner that is phase-locked to the hippocampal θ rhythm *in vivo* (Brazhnik and Fox, 1997), although given the slow kinetics of mAChR activation and the bulk or volume transmission that probably characterizes most ACh release, the cholinergic cells are unlikely to be true pacemakers for θ rhythms.

Higher frequency γ oscillations in the hippocampus may act in concert with θ oscillations to encode and retrieve memory traces (Bragin et al., 1995; Csicsvari et al., 2003). γ and θ can occur concurrently, particularly in deeper hippocampal layers, and γ is strongest during periods of θ (Bragin et al., 1995; Buzsáki, 2005). A cross-frequency correlation (CFC) analysis showed that the degree of θ–γ coupling in CA3 *in vivo* increased during a context learning task in rats, and the strength of the coupling was directly correlated with the increase in performance accuracy (Tort et al., 2009). Their interaction provides a mechanism for the temporal ordering of individual episodic events (θ) and the reconstruction of different facets of a memory (γ). The latter, the so-called “binding phenomenon”, occurs when disparate cortical areas encoding different facets of a memory, such as the shape, color, and texture of an object, must be activated simultaneously in order to form a coherent representation of the object (Singer and Gray, 1995). However, the hypothesis that neural synchrony through coherent γ oscillation solves the binding problem is controversial. γ rhythms have also been proposed to provide the exact temporal framework for spike-timing-dependent plasticity to occur, as θ oscillations would be too slow for the rapid and precise coordination required (Axmacher et al., 2006).

GABA inhibition is widely agreed to be a major factor in the generation of γ (Whittington and Traub, 2003; Whittington et al., 2011). Nevertheless, the details of the connection between endogenous ACh in hippocampus are less clear for γ than for θ rhythms. Atropine reduces hippocampal γ power in awake, behaving animals (Leung, 1985; Hentschke et al., 2007), and reduces θ–γ coupling (Hentschke et al., 2007). *In vivo*, however, hippocampal γ is abolished by lesioning the entorhinal cortex (Buzsáki, 2002, 2005), suggesting a requirement for glutamatergic, but not cholinergic, inputs in the generation of γ. After this ablation a somewhat slower γ appears in CA3 and CA1, suggesting that, under some conditions, the hippocampus can generate a form of γ without the extrinsic glutamatergic inputs from the entorhinal cortex. One hypothesis is that ACh could trigger the intrinsic γ oscillations: γ can be pharmacologically-induced by CCh in hippocampal slices (Fisahn et al., 1998; Traub et al., 2003). Muscarine-induced γ rhythm in the CA3 region *in vitro* depends on the activation of M1 mAChRs in PCs, and is absent in M1 mAChR^−/–^ mice. This M1-dependent γ is produced by modulation of the mixed-cation Na^+^/K^+^ current and the Ca^2+^ -dependent non-selective cation current, but does not involve modulation of the M-current (Fisahn et al., 2002).

Although ACh-induced oscillations are prominent in the hippocampus, other brain regions can generate them locally as well, especially the neocortex. Stimulus-evoked γ activity in visual cortex is blocked by intracortical infusion of atropine (Rodriguez et al., 2004), for example. Unlike the MS/DBB cholinergic projection, which drives primarily the lower frequency θ oscillations in the hippocampus, the NBM is believed to underlie cortical “activation”, or a decrease in lower frequency synchronized EEG activity accompanied by an increase in local γ frequency. Such a mechanism may underlie, among other things, selective attention (review by Wang, 2010). The discharge rate of NBM cholinergic neurons is much higher during cortical activation, ACh release in the cortex is higher, and lesions of NBM decrease both cortical ACh release and cortical activation (Dringenberg and Vanderwolf, 1998). Despite a lack of direct projections to the hippocampus, activity in the NBM does affect hippocampal activity, and lesions of the NBM can modulate event-related oscillations in the delta (δ, 0.1–3 Hz), θ, β (15–29 Hz), and γ frequency ranges in dorsal hippocampus, as well as in the amygdala and pre-frontal cortex (PFC). Cholinergic neurons in the MS/DBB and NBM could modulate oscillations between and within brain regions, respectively—excitotoxic lesions of MS/DBB decrease γ frequency event-related oscillations in frontal cortex, and reduce phase locking between frontal cortex and hippocampus in the θ band (Sanchez-Alavez et al., 2014). Similar lesions of NBM cause increases in frontal cortex δ and θ, decreases in γ, and reductions in phase-locking between frontal cortex and hippocampus in the γ band. The NBM mediates increases in cortical δ activity during stress in the PFC (a direct target) and retrosplenial cortex (an indirect target; Knox and Berntson, 2008).

### *In vitro* models of ACh-generated oscillations in hippocampus and neocortex

Whether ACh plays a causal or modulatory role in rhythm generation *in vivo* is controversial. *In vitro*, cholinergic agonists or released ACh generate rhythmic cell firing, synaptic currents, and local field potentials (LFPs). These effects have been reported most frequently in hippocampal slices, but also occur in neocortical slices. Thus, the brain slice preparation has been an invaluable tool for studying the mechanisms by which ACh can generate oscillations at multiple levels, especially when considering the “inverse problem” of the LFP (Buzsáki et al., 2012), i.e., the task of inferring microscopic variables (e.g., synaptic or cellular components) from macroscopic data (e.g., a current source density analysis). Solving the “forward problem” i.e., identifying the synaptic or non-synaptic events generating the LFP by correlating them with the LFP, may be a prerequisite for solving the inverse problem.

**Figure 2:**
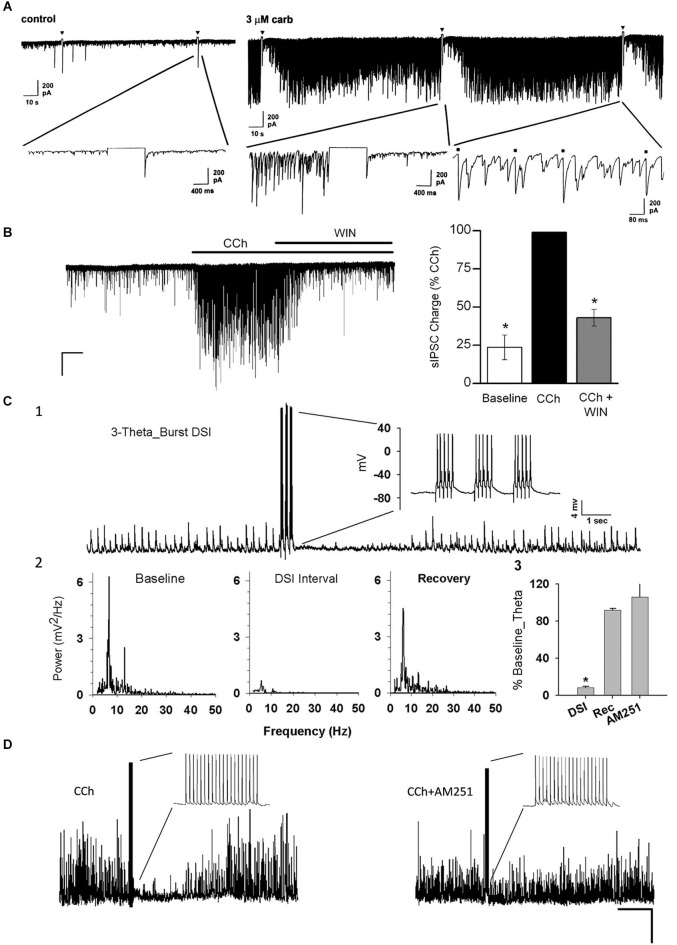
**Carbachol (CCh) elicits persistent occurrence of large, rhythmic inhibitory synaptic responses in hippocampus and neocortex. (A)** Representative whole-cell recording from a Sprague/Dawley rat CA1 pyramidal cell (PC) in control saline (iGluR antagonists present in experiments shown in all panels) *in vitro* and a 1-s depolarizing voltage-step (downward triangles) has no effect on the small spontaneous IPSCs visible on the baseline. Bath application of 3 μM CCh induces a persistent barrage of large IPSCs that are transiently interrupted by the periods of DSI that occur after the voltage steps (From Martin and Alger, 1999; with permission). **(B)** Representative trace from a layer II/III neocortical PC from a Swiss CD-1 mouse slice. A large barrage of IPSCs occurs after CCh (5 μM) is added to the bath, and the IPSCs are suppressed by further addition of the CB1R agonist WIN55212-2 (5 μM). From Trettel et al. (2004) with permission. **(C1)** Representative sharp electrode recording of large, rhythmic IPSPs induced by 5 μM CCh in a rat CA1 PC in a hippocampal slice. Brief bursts of action potentials induced by depolarizing current injections induced a period of DSI. **(C2)** Power spectral analyses of the IPSPs before, during and after DSI. Note peak power in the theta frequency range. **(C3)** Group data showing “relative theta power” (integral of power from 4–14 Hz/total power from 2–50 Hz) from experiments as in **C1**, **C2**. DSI strongly suppressed the theta power (which recovered fully following DSI). DSI was abolished by the CB1R antagonist, AM251 (3 μM). From Reich et al. (2005) with permission. **(D)** DSI of 5 μM, CCh-induced IPSPs produced by an action potential train in a layer II/III PC in a mouse neocortical slice. DSI was abolished by 5 μM AM251. From Trettel et al. (2004) with permission.

Application of CCh to hippocampal slices induces θ frequency membrane potential oscillations and firing in a majority of cells in CA1, CA3, and DG (Bland et al., 1988). The mixed ACh agonist CCh induces oscillations in the LFP (Hájos et al., 2004), membrane potentials or firing patterns (Williams and Kauer, 1997), and rhythmic inhibitory post-synaptic responses in CA1 PCs (Reich et al., 2005). It has been proposed that ACh-generated rhythms are initiated in CA3 and transmitted into CA1 via the Schaffer collaterals (Williams and Kauer, 1997; Fisahn et al., 1998; Buzsáki, 2002), although using a novel slicing procedure, Pietersen et al. (2014) report evidence for intrinsic γ generation in CA1. *In vivo* Type II (atropine sensitive) θ might additionally require rhythmic inhibition onto interneurons from septal GABAergic afferents (Stewart and Fox, 1990; Tóth et al., 1997; Buzsáki, 2002). These models cannot explain all of the data however, since θ-frequency rhythmic sIPSP/Cs in CA1 PCs can be induced by CCh application to slices in the presence of iGluR antagonists (Figure 2C; Reich et al., 2005; Karson et al., 2008) or in small isolated sections of CA1 (Reich et al., 2005).

Rhythmic inhibition is thought to be essential for the generation of hippocampal oscillations, including ACh-mediated oscillations (Stewart and Fox, 1990; Buzsáki, 2002; Mann and Paulsen, 2007; Klausberger and Somogyi, 2008). Electrical stimulation of cholinergic afferents in hippocampal slices increases the frequency of spontaneous IPSPs in CA1 PCs (Pitler and Alger, 1992b). Stimulation of single hippocampal BCs at θ frequency produces unitary IPSPs in synaptically connected PCs that are sufficient to entrain the PC firing (Cobb et al., 1995). As noted earlier, most interneuron types in CA1 are modulated by ACh (McQuiston and Madison, 1999a,b; Widmer et al., 2006). Hippocampal interneurons are very heterogeneous, and different interneuron classes will fire in a distinct pattern (or not at all) within a given type of oscillation (Klausberger et al., 2003).

The two populations of perisomatic-targeting interneurons, PV+ and CCK+ BCs, are activated by ACh (Karson et al., 2009; Cea-del Rio et al., 2010), and have been functionally implicated in fast and slow oscillations, respectively, generated by cholinergic agonists (Reich et al., 2005; Gulyás et al., 2010). Fast, iGluR-mediated excitatory stimulation of PV+ cells produces IPSPs that contribute to atropine-insensitive, but not atropine-sensitive θ rhythms (Korotkova et al., 2010). Exogenous muscarinic agonists or endogenous ACh activate PV+ cells (Cea-del Rio et al., 2010). At the same time, M2 mAChR activation suppresses GABA release from PV+ terminals, but does not entirely eliminate it (Hájos et al., 1998; Gulyás et al., 2010). Application of CCh, or release of endogenous ACh, generates θ frequency IPSP/Cs in CA1 PCs which can be disrupted by exogenous or endogenous cannabinoids, suggesting that ACh generates θ in CA1 by activating CCK+ BCs (discussed below, and cf Cea-del Rio et al., 2012). In addition to the perisomatic-targeting interneurons, the dendritic targeting O-LM cells exhibit rhythmic membrane potential oscillations in response to CCh application, even in the absence of fast glutamatergic signaling (Chapman and Lacaille, 1999; Lawrence, 2008).

In CA3, CCh application generates γ-frequency oscillations (Hájos et al., 2004; Oren et al., 2006, 2010). The γ LFPs are greatly suppressed by a μOR agonist (μORs are predominantly located on PV+ terminals, where their activation suppresses GABA release), and PV+ BCs show the highest degree of phase modulation by the LFP (Gulyás et al., 2010). *In vitro*, morphine also suppresses CA3 γ rhythms that arise from tetanic stimulation of the s. oriens, and this effect is blocked by the μOR antagonist cyprodime (Whittington et al., 1998). Thus, it would appear that in CA3, unlike in CA1, ACh generates oscillations by selectively activating the PV+ network.

Whereas θ and γ are the two prominent ACh-generated oscillations in hippocampus, several different frequency ranges have been observed in neocortical slices. Unlike hippocampal slices, where atropine-sensitive θ or γ oscillations can be reliably elicited by CCh alone, oscillations in neocortical slices are frequently generated by applying kainate, or GABA-A receptor antagonists along with CCh. For example, CCh application generates β oscillations in rat PFC (van Aerde et al., 2009). The combination of CCh plus kainate also elicits β oscillations in primary motor cortical slices that are unaffected by AMPA blockers but prevented by GABA-A or a gap junction blocker (carbenoxolone) (Yamawaki et al., 2008). CCh generates β activity in an intact preparation of newborn rat neocortex that is dependent on mAChRs and AMPA/kainate receptors, but not GABA-A receptors (Kilb and Luhmann, 2003). CCh plus kainate elicits γ in somatosensory and visual cortex slices (Oke et al., 2010). Both CCh and bicuculline application are required to generate oscillations in neonatal rat cortical slices (Lukatch and MacIver, 1997). CCh and bicuculline also generate 3–22 Hz LFP activity in slices of occipital lobe, including “spiral” waves (Huang and Hsu, 2010). Thus, the differences in frequency ranges and pharmacology of ACh generated oscillations in neocortical slices might reflect the different circuitry activated by ACh in various cortical regions.

## eCBs and mAChRs

Depolarization-induced suppression of inhibition (DSI) is profound, reversible disinhibition of principal cells that was initially described in the hippocampus as a transient suppression of sIPSPs or sIPSCs that followed a brief, 1 or 2 s, depolarizing current injection into a CA1 PC (Pitler and Alger, 1992a). A great deal of evidence showed that DSI was mediated by a retrograde signal process, i.e., as a result of Ca^2+^ entry into a PC, a chemical messenger was released and traveled backwards across the synaptic cleft, and by activating an initially unidentified G-protein coupled receptor on certain GABAergic nerve terminals, temporarily prevented GABA release (Alger, 2002, for review). CCh markedly increased IPSP frequency by activating mAChRs on hippocampal interneurons (Pitler and Alger, 1992b) and in addition enhanced and prolonged DSI (Pitler and Alger, 1992a). nAChRs were found to have no role in enhancing DSI (Martin and Alger, 1999): nicotine did not mimic the effects of CCh and a broad spectrum nAChR-antagonist, mecamylamine, did not antagonize them. In contrast, the CCh-effects on DSI were abolished by atropine and other mAChR antagonists, and were mimicked by selective mAChR agonists, such as Oxo-M. A battery of mAChR antagonists, including pirenzepine, 4-DAMP, and AFDX-116, led to the conclusion that either the M1 or M3, but not the M2 receptor, were responsible for inducing persistent action potential firing of the interneurons that were most highly sensitive to DSI (Martin and Alger, 1999; cf Trettel et al., 2004), i.e., the firing of these interneurons produced GABAergic IPSCs that were readily suppressed by DSI (Figure 2). The results explained the great sensitivity of CCh-induced sIPSCs to DSI, but provided no insight into the actual mechanism of DSI itself.

In 2001 the retrograde messenger for DSI was reported to be an eCB, and the GPCR-coupled receptor on the interneuron terminals was the cannabinoid receptor, CB1R (Ohno-Shosaku et al., 2001; Wilson and Nicoll, 2001). There are two major eCBs, anandamide and 2-arachidonyl glycerol (2-AG), and 2-AG was demonstrated to be the main signaling eCB (see Kano et al., 2009 for review and Figure 1 DSI). It was soon found that a mGluR agonist, (±)-1-aminocyclopentane-trans-1,3-dicarboxylic acid (ACPD) or the selective group I mGluR agonist, (S)-3,5-dihydroxyphenylglycine (DHPG), markedly increased DSI (Varma et al., 2001). At low concentrations, DHPG enhanced DSI without affecting the IPSCs directly, while at higher concentrations DHPG directly suppressed the IPSCs as well. Most significantly, the three phenomena, DSI, the enhancement of DSI by mGluRs, and the direct suppression of IPSCs by high concentrations of an mGluR agonist were all abolished by a CB1R antagonist, and absent in the CB1R^−/–^ mouse. The explanation was that mGluRs on PCs either enhanced the mobilization of eCBs by DSI, or directly caused eCB mobilization from these cells, and the eCBs crossed the synaptic cleft and inhibited the IPSCs by activating the CB1Rs on the interneuron terminals (Figure 1; “mobilized” is the preferred term because the processes of eCB synthesis and release cannot be distinguished electrophysiologically and are not inextricably linked, see Alger and Kim, 2011, for review). eCBs are also retrograde signals at excitatory synapses (Kreitzer and Regher, 2001) and mGluRs mobilize eCBs there as well (Maejima et al., 2001). Thus eCBs are not only produced by high levels of Ca^2+^ in principal cells, but are intermediaries in modulating synaptic transmitter release by glutamate, and hence were likely to have a broad range of actions.

Both group I mGluRs and M1-class mAChRs are GPCRs that are coupled to G_q/11_ type G-proteins. Kim et al. (2002) found that activating mAChRs with low μM concentrations of CCh markedly enhanced DSI without affecting the IPSCs directly, but at higher concentrations directly suppressed them. For concentrations up to ~5 μM CCh, the suppressive effects on the IPSCs were entirely reversed by a CB1R antagonist, demonstrating that, like the type I mGluRs, mAChRs could mobilize eCBs. Above 5 μM, a portion of the CCh-induced IPSC suppression could not be prevented by CB1R antagonists, suggesting that a distinct, eCB-independent form of synaptic depression also occurred. Significantly, bath-application of the AChE inhibitor, physostigmine, in the absence of other treatments, induced an atropine- and CB1R-dependent suppression of IPSCs, indicating that the low, tonic levels of ACh present in hippocampal slices were sufficient to induce persistent mobilization of eCBs (Kim et al., 2002). In accordance with this suggestion, Colgin et al. (2003) found that an AChE inhibitor depressed fEPSPs in the dentate gyrus and CA1 of hippocampal slices. This effect was absent when, prior to the *in vitro* experiments, the fimbria/fornix was lesioned and allowed to deteriorate. Most importantly, the effect was abolished by atropine and a CB1R antagonist, but unaffected by an M2 mAChR inhibitor, clearly arguing that ACh from cholinergic afferents could suppress glutamate transmission heterosynaptically via mAChR-induced, eCB release. Presumably *in vivo* release of ACh can have the same ability to regulate synaptic transmission indirectly by stimulating the release of eCBs. It is important to note that, in addition to glutamate and GABA, eCBs may also directly regulate the release of ACh itself (Gifford and Ashby, 1996; Kathmann et al., 2001; Tzavara et al., 2003; Degroot et al., 2006), although detailed physiological mechanisms of these effects have yet to be worked out.

Neither the mAChR-dependent increase of DSI, nor the direct mobilization of eCBs by mAChR activation was associated with any change in [Ca^2+^]_i_ (Kim et al., 2002), suggesting that the GPCR-dependent pathway of eCB mobilization and the Ca^2+^ -dependent pathways were independent. Indeed, the ability of mAChRs to mobilize eCBs was occluded when GTP_γ_S, a generalized activator of G-proteins, was infused into the cells, but unaffected when intracellular Ca^2+^ was chelated by high concentrations of intracellular BAPTA (Kim et al., 2002). Hence, mAChR-dependent eCB mobilization is independent of changes in [Ca^2+^]_i_ but entirely dependent on G-protein activation, whereas, conversely, DSI is totally dependent on a rise in [Ca^2+^]_i_ and unaffected by GTP_γ_S. Thus the two pathways for eCB mobilization are independent, but, importantly, can interact, as shown by the enhancement of DSI (Ca^2+^-dependent pathway) by co-activation of a GPCR pathway (Varma et al., 2001; Kim et al., 2002).

The findings on mAChRs and eCBs were confirmed and extended in paired recordings from principal cells and interneurons in tissue-cultured primary hippocampal neurons (Ohno-Shosaku et al., 2003). Ohno-Shosaku et al. (2003) observed no real change in the ability of CCh (or Oxo-M) to mobilize eCBs in tissue cultured cells from knock out mice with either M1^−/–^ or M3^−/–^ mAChRs eliminated, but a virtual elimination of the eCB-related effects in the combined M1^−/–^/M3^−/–^ line. This demonstrated involvement of both M1 and M3 mAChRs, and suggested that activation of either receptor alone could produce enough eCBs for maximal suppression of IPSCs. In the dorsal striatum, tonic activity of the cholinergic interneurons leads to a persistent enhancement of DSI in the medium spiny neurons, which is blocked by the M1 antagonist, pirenzepine, and is absent in the M1^−/–^ mouse, and hence is also mediated via M1 mAChRs (Narushima et al., 2007).

Although these issues have not been dissected as thoroughly in the neocortex as in the hippocampus, the apparently identical observations of IPSC frequency enhancement by CCh and eCB-dependent DSI in neocortical slices (Figure 2; cf. Fortin et al., 2004; Trettel et al., 2004; Yoshino et al., 2011) makes it likely that the association between M1/M3 receptors and eCBs holds there as well. Fukudome et al. (2004) showed, also in paired principal cell-interneuron recordings in hippocampal tissue-culture, that the *eCB-independent*, CCh-induced suppression of GABA release was mediated by M2 receptors, as it was mimicked by the M2 preferring agonist, gallamine, and absent in tissue from the M2^−/–^ mouse. Importantly, the M2-mediated suppression occurred in those interneurons that were not sensitive to suppression by eCBs, and vice versa, interneurons from which GABA release was suppressed by eCBs were insensitive to suppression by gallamine. It is therefore likely that the interneurons from which GABA release is inhibited indirectly by M1/M3 (i.e., eCB-sensitive) actions and those inhibited by M2 mAChRs are of different classes. Undoubtedly, the former represented the CCK+/CB1R+ interneurons and the latter the CCK−/CB1R− interneurons, probably the PV+ cells, although the cells were not immunologically identified. The results from slices (Martin and Alger, 1999; Kim et al., 2002) and tissue-culture (Ohno-Shosaku et al., 2003; Fukudome et al., 2004) are in substantial agreement in identifying the M1/M3 receptors as the likely stimulants of the eCB mobilization, while M2 receptors mediate an eCB-independent form of presynaptic inhibition.

Studies with tissue from phospholipase C beta (PLC_β_) isoform-specific, knock-out mice, ${\text{PLC}}_{\text{β1}}^{\text{−/−}}$ and ${\text{PLC}}_{\text{β4}}^{\text{−/−}}$ showed that PLC_β_ is an essential element in the G-protein signaling pathway between mAChRs, or mGluRs, and eCBs in hippocampus and cerebellum, with the different isoforms being predominant in different brain structures (Hashimotodani et al., 2005; Maejima et al., 2005); in the absence of PLC_β_ neither of these GPCRs can mobilize eCBs. Another key observation was that DSI is independent of PLC_β_ (Hashimotodani et al., 2005), which confirms that the Ca^2+^ -dependent and GPCR-dependent forms of eCB mobilization utilize distinct biochemical pathways, although 2-AG is the eCB produced by both of them. PLC_β_ is activated by M1/M3 mAChRs and requires Ca^2+^ for its enzymatic activity, hence it is proposed that PLC_β_ acts as a coincidence-detector (Hashimotodani et al., 2005) that can integrate the actions of Ca^2+^ and G-proteins and thereby explain the ability of mAChRs to enhance DSI. Some challenges to this straightforward story exist (Edwards et al., 2006, 2008) and future work should provide more mechanistic detail. Nevertheless, PLC is a part of the molecular cascade that produces 2-AG, though not anandamide, and so the involvement of PLC also confirmed that 2-AG was the major eCB produced by mAChRs. 2-AG is produced by the enzyme diacylglycerol lipase alpha (DGL_α_) and the absence of this enzyme in ${\text{DGL}}_{\text{α}}^{\text{−/−}}$ mice prevents the mAChRs from mobilizing eCBs (Tanimura et al., 2010; Yoshino et al., 2011). DSI is also absent in DGLα^−/−^ mice (Gao et al., 2010; Tanimura et al., 2010; Yoshino et al., 2011), unlike the case with PLC_β_^−/−^ mice in which DSI is unaffected. Evidently, if diacylglycerol is the common precursor for the production of 2-AG, two independent pathways supply diacylglycerol to DGL_α_ for the production of 2-AG.

The summary picture is that M1 and M3 mAChRs mobilize the eCB 2-AG via a molecular pathway involving PLC_β_ and DGL_α_.

## Optogenetic studies

**Figure 3:**
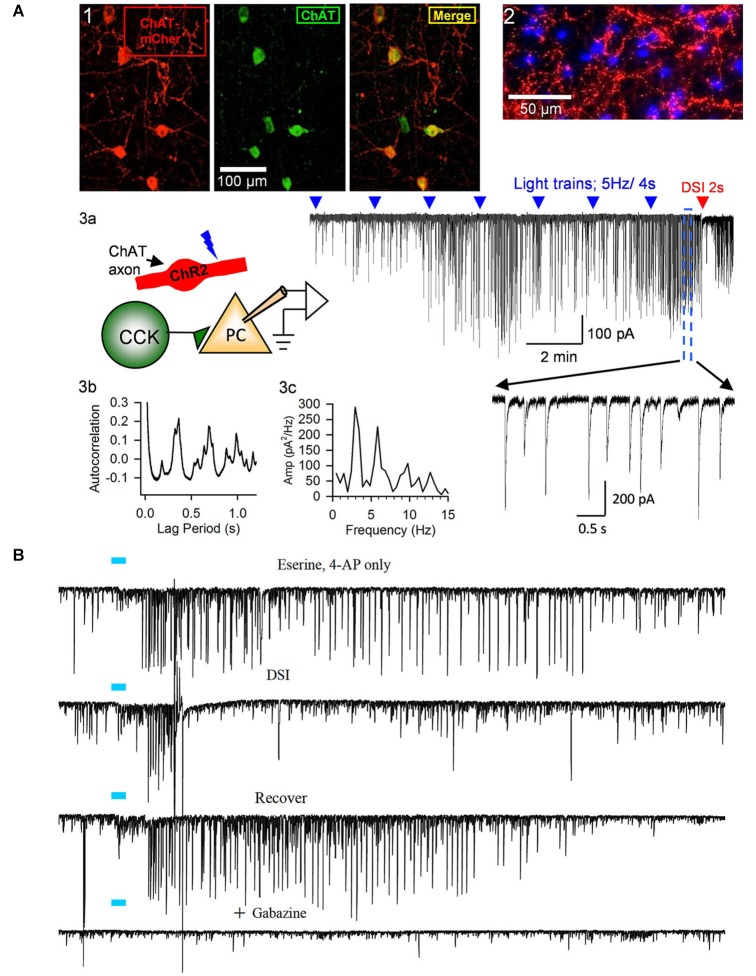
**Release of ACh by light-stimulation of ChR2 in ChAT-expressing axons induces bursts of rhythmic IPSCs in the CA1 region of hippocampal slices. (A1)** Examples of ChAT-positive cells in MS/DBB expressing ChR2+mCherry following viral injection of AAV (see text) into a ChAT-Cre mouse (from Nagode et al., 2011, with permission), and **(A2)** ChR2+mCherry axons plus DAPi staining showing cholinergic axons in proximity to cells in CA1. Details of procedures are found in Nagode et al. (2011). **(A3)** Diagram of experimental setup; light-stimulation of ChR2-expressing axons in CA1 release ACh onto CCK+ interneurons that fire trains of action potentials and thereby induce IPSPs in CA1 PCs. Sample trace to the right shows trains of blue-light pulses (blue triangles) given at 2 min intervals gradually come to induce prolonged bursts of GABAergic IPSCs (downward deflections in the presence of iGluR blockers to prevent EPSC occurrence in experiments shown in this panel; cf. expanded portion, below) in a PC. A 2-s voltage step was given to the PC near the end of the trace (red arrow) to induce DSI, the transient interruption of the IPSCs. **(A3b,c)** Autocorrelation function and power spectrum of data from this cell illustrate the rhythmic nature of the ACh-induced IPSCs. Neither physostigmine nor 4-AP were used in this experiment. **(B)** Top trace, light pulse (blue bar) delivered to ChR2-expressing axons in a slice from a ChAT-Cre, AAV-injected mouse induced a burst of large IPSCs in a CA1 PC; second trace, the burst of IPSCs was interrupted during the period of DSI produced by a brief depolarization of the PC; third trace, recovery of the IPSC burst after the DSI trial; fourth trace, application of the GABA-A receptor antagonist, gabazine, blocks all light-induced activity, confirming their identity as IPSCs. Physostigmine, 1 μM, and 4-AP, 20 μM, were present in the bathing solution. Results are typical of numerous experiments. **(A2, B)** from D.A. Nagode Ph.D. thesis at http://archive.hshsl.umaryland.edu/handle/10713/2315. **(A3a–c)** is a typical result (c.f. Nagode et al., 2011).

Release of endogenous ACh via bulk tissue electrical stimulation activates interneurons in hippocampus (Pitler and Alger, 1992b; Widmer et al., 2006), and drives inhibitory oscillations in CA1 PCs (Martin and Alger, 1999). However, bulk stimulation of tissue can also affect non-cholinergic fibers and glia, and complete pharmacological isolation of ACh responses is often not possible. The advent of optogenetics has allowed for stimulation or silencing of specific neuron populations in slice preparations as well as *in vivo*, and the cholinergic system was one of the first targeted for optogenetic manipulation (see Fenno et al., 2011, for review). Expression of the light-activated non-selective cation channel, Channelrhodopsin2 (ChR2, Boyden et al., 2005), in cholinergic neurons allows for specific stimulation of cholinergic cells or fibers and release of endogenous ACh in slice preparations. ChR2 is commonly delivered *in vivo* to target nuclei via an adeno-associated virus (AAV) vector, which has a high tropism for neural tissue and can result in expression levels exceeding 90% in target cells (Figure 3). To ensure specificity of ChR2 expression in cholinergic cells, the cre-loxP system has been utilized: the vector constructs carry a double-floxed inverted (FLEXED) ChR2 sequence (Atasoy et al., 2008), and the vectors are injected into the brains of ChAT-Cre mice, which express cre recombinase only in cholinergic cells. Injection of AAV-ChR2 into cholinergic nuclei results in expression of ChR2 in distal axon terminals of projection neurons in ~2–5 weeks (Figure 3A; cf Gu and Yakel, 2011; Nagode et al., 2011, 2014; Tang et al., 2011; Kalmbach et al., 2012; Kalmbach and Waters, 2014), allowing for stimulation of ACh release in slice preparations which do not retain the cholinergic cell bodies (e.g., the hippocampus). The variability in expression time may have to do with differences in viral serotype used (AAV2/1—2/9 have all been used), viral titer (typically higher than 10^12^ genome copies/ml), or ChR2 variant. In general the AAVs appear to have equal tropism for all cells, although selective transformation of inhibitory cells in the cortex was reported with low titer levels (e.g., Nathanson et al., 2009) and specific tropism for cholinergic neurons has apparently not been reported. AAV2/1, 2/5, and 2/9 have all been used successfully with the cre/lox strategy in the Chat-Cre mice. The ChR2-variant, ChIEF, which has faster kinetics, hence greater suitability for high frequency stimulation, and increased steady-state photocurrent, than does the more commonly used H134R ChR2, has also been targeted to septal cholinergic cells and used to stimulate ACh release in hippocampal slices (Bell et al., 2011, 2013). ChIEF appears to show superior expression and transport to plasma membranes when compared to H134R ChR2 (Mattis et al., 2011), although whether this translates into advantages of ChIEF over H134R ChR2 for release of ACh from terminals is not yet known.

In addition to the viral strategy, transgenic mouse lines constitutively expressing ChR2 have been developed. The most widely-used strain is the ChAT-ChR2-EYFP mouse (Strain 014546; Jackson Laboratories; e.g., Ren et al., 2011; Pinto et al., 2013). *In vivo* stimulation of basal forebrain ACh neurons, or their axon terminals in visual cortex, has also been achieved using this mouse. Pinto et al. (2013) found that ChR2 activation of basal forebrain cell bodies or axon terminals desynchronized cortical activity and enhanced visual discrimination. On the other hand, crossing Chat-Cre mice with the inhibitory halorhodopsin or archerhodopsin-expressing mice to silence basal forebrain cholinergic neurons synchronized cortical activity and decreased visual discrimination. However, the ChAT-ChR2-EYFP strain exhibits some deficits in attention and working and spatial memory, due to increased copy number and expression of VAChT (Kolisnyk et al., 2013), which should be considered before using them, especially for behavioral studies. Indeed, because efficiency of optogenetic activators or silencers is low, the high protein expression required to affect neuronal activity, especially during brain development, might permanently alter brain circuitry and therefore behavior. The expression of opsins in ChAT-Cre mice also varies across brain regions. The viral transduction method in adults, though more invasive, may be more advantageous than constitutively expressing mouse models in some cases.

It must be noted that in some parts of the nervous system ACh is reportedly co-localized with other neurotransmitters, including glutamate (e.g., Allen et al., 2006; Lamotte d’Incamps and Ascher, 2008) and GABA (Bayraktar et al., 1997, but see Chédotal et al., 1994), and furthermore that they may be co-released with ACh (e.g., Allen et al., 2006; Lamotte d’Incamps and Ascher, 2008; Ren et al., 2011). In principle co-release of glutamate by ChR2-induced depolarization could confound studies of optogenetic ACh release. Although we have seen no evidence for this in our experiments (Nagode et al., 2011, 2014), the possibility should be explored. Therefore, even precise cellular targeting of light-activated molecules to ChAT-expressing cells may not absolutely guarantee that light stimulation will cause the release of only ACh, or conversely, that any light-induced biological effects can unambiguously be attributed to ACh *a priori*. This will probably be true whether the opsins are expressed in the target cells virally or transgenically. Additional pharmacological or perhaps molecular biological controls would have to be taken to identify the active agent. For the use of pharmacological tools, *in vitro* slice preparations will undoubtedly be most effective. Of course, from the point of view of behavioral relevance, *in vivo* preparations will be most desirable. Thus, it seems that combinations of *in vivo* and *in vitro* approaches will be required to achieve definitive conclusions regarding axonally released transmitter actions, even using optogenetic techniques, for the forseeable future.

**Figure 4:**
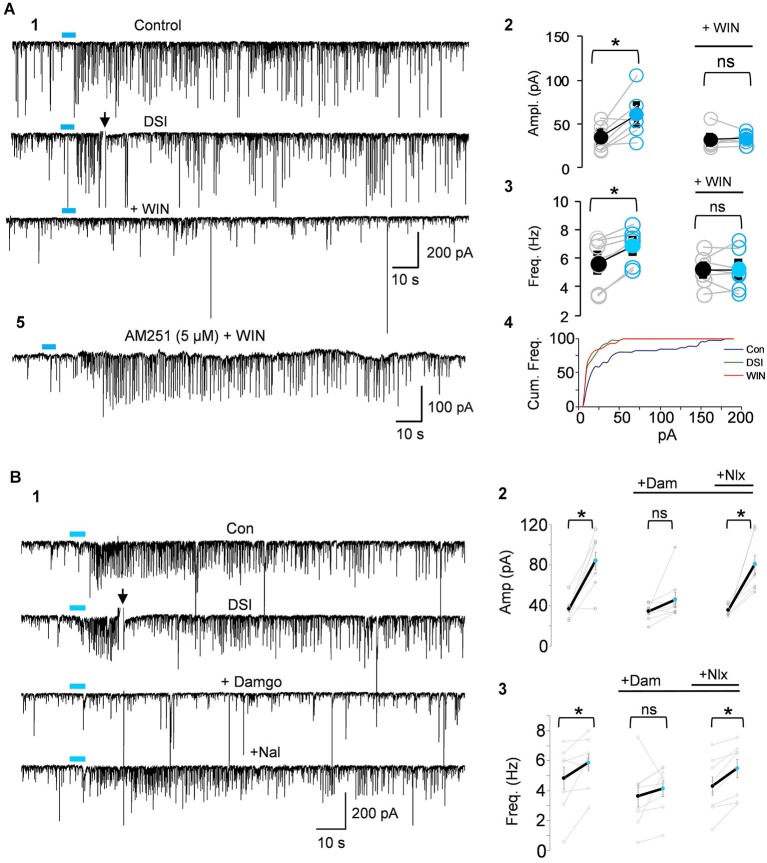
**IPSCs triggered by light-induced ACh release arise from CB1R+ interneurons. (A1)** Top two traces as in Panel Panel 3B; the CB1R agonist, WIN55212-2 (WIN) was then applied to the bathing solution of the same cell, and prevented the ability of ACh to induce repetitive IPSCs. **(A2–A4)** Group data showing that the increases in IPSC amplitudes **(A2)** or frequency **(A3)** or cumulative frequency **(A4)** induced by light in control solution (left graphs in **A2,A3**), were occluded in WIN-treated slices; i.e., that they arose from CB1R+ interneurons. **(A5)** Shows that pretreatment with the CB1R antagonist, AM251, prevented the ability of WIN to suppress the ACh-induced IPSCs. **(B1)** DSI-sensitive IPSCs induced by ACh release can also be reversibly suppressed by the μOR agonist, DAMGO, and recover when the μOR antagonist, naloxone, is applied. **(B2,3)** Show group data for experiments such as in **(A)**. Physostigmine, 1 μM, and 4-AP, 5–20 μM, were present in the bathing solution. Figures taken from D. Nagode Ph.D. Thesis, at http://archive.hshsl.umaryland.edu/handle/10713/2315.

For the study of ACh effects in slice preparations, particularly oscillations or other network phenomena, generating sufficient ACh release is a significant concern. ChR2 expression in axon terminals is usually weaker than in somata, and axons from the basal nuclei are unavoidably severed by the slicing. If the “bulk transmission” hypothesis is correct (Vizi and Kiss, 1998), large amounts of ACh release might be required to activate receptors on target cells, especially since acetylcholinesterase (AChE) hydrolyzes ACh very efficiently. It is nevertheless possible to stimulate long bursts of DSI-sensitive IPSCs that closely mimic those induced by mAChR agonists (e.g., Figures 3, 4). While these IPSCs can be elicited by light-induced ACh release in slices in normal recording saline (Figure 3A), we (Tang et al., 2011; Nagode et al., 2011, 2014) often use physostigmine to inhibit AChE, and a low concentration of the K-channel blocker, 4-AP, to enhance ChR2-induced ACh release (as has been done by others, e.g., Petreanu et al., 2007; Hull et al., 2009). This greatly increases the occurrence of ChR2-induced rhythmic activity without otherwise altering the IPSCs (Figures 2B and 3; cf Nagode et al., 2011). Such pharmacological enhancement is not necessary for evoking post-synaptic ACh currents (Bell et al., 2011, 2013) or nAChR-dependent plasticity of EPSCs in CA1 PCs (Gu and Yakel, 2011), suggesting that less ACh is required to generate single cell firing than sustained network activity.

An important new finding (Nagode et al., 2014) was that light-induced ACh release triggers IPSCs that are sensitive to DSI, and that most of them are also sensitive to μOR agonists even when the output of PV+ cells (which express the great majority of hippocampal μORs) has been abolished (Figure 4B). Dual CB1R/μOR sensitivity has been reported (e.g., Neu et al., 2007; Glickfeld et al., 2008) but it was surprising to encounter it so frequently in the optogenetic experiments. The explanation for this observation is not understood, but may imply that axonally released ACh has an unexpectedly strong tendency to activate dually sensitive interneurons, which could be important for understanding cannabinoid/opioid interactions *in vivo*. It will be of great interest to explore the effects of silencing septal cholinergic neurons with halorhodopsin or archerhodopsin *in vivo* during ongoing hippocampal θ oscillations. The unique ability to release ACh from cholinergic axons optogenetically probably made this discovery possible.

While dramatic effects of optogentically released ACh are on the induction of θ rhythm frequency oscillations of IPSPs via activation of mAChRs, a pulse of released ACh also elicits a burst of IPSCs by activating nAChRs and a highly novel mechanism involving T-type Ca^2+^ channel activation and Ca^2+^ stores (Tang et al., 2011). These events occur even in the presence of TTX, strongly suggesting that the nAChRs are on the GABAergic nerve terminals. Moreover, since there are no morphologically defined synapses along these axons, this appears to be a direct example of a non-synaptic effect of axonally released ACh. Non-synaptic stimulation of GABA release can also be produced by optogenetic stimulation of striatal cholinergic interneurons. Interestingly, the same ionic mechanism as proposed in hippocampus (Tang et al., 2011) appears to operate in striatum (Nelson et al., 2014).

## mAChRs, eCBs, and neuronal network oscillations in hippocampus

mAChR-activation induces the firing of CCK+/CB1R+ cells and IPSCs from these cells are a major factor in inhibitory θ rhythms. However, there is extremely good evidence that the M1 and M3 mAChRs are also very effective in stimulating the mobilization of eCBs. One might expect that the GABAergic output from CCK+/CB1R+ cells would be rapidly eliminated by the eCBs, and indeed it has been proposed that CCh-generated eCBs silence the CB1R+ cells during rhythm generation in CA3 (Gulyás et al., 2010; Holderith et al., 2011). Nevertheless, on the contrary, the CCh-induced IPSCs are highly susceptible to inhibition by DSI (e.g., Pitler and Alger, 1992b; Alger et al., 1996; Martin and Alger, 1999; Wilson and Nicoll, 2001; Kim et al., 2002; Hampson et al., 2003; Fortin et al., 2004; Trettel et al., 2004; Yoshino et al., 2011). Thus the CB1R+ interneurons are not entirely silenced by the mAChR-induced eCBs. As noted, studies from DGLα^−/–^ mice, which are incapable of generating the major eCB, 2-AG, confirm that both mAChR-dependent eCB effects and DSI are mediated by 2-AG (Tanimura et al., 2010; Yoshino et al., 2011), so differences in eCB identity cannot account for the continued sensitivity of CCh-induced θ IPSCs to DSI.

An entirely different mechanism was described by Makara et al. (2007) who reported that, in the presence of CCh, the eCB-system becomes dependent on nitric oxide (NO) production. When mAChRs were activated DSI could be prevented by inhibitors of NO synthesis or NO scavengers. NO scavengers injected into the postsynaptic PCs prevented the action of NO and soluble cGMP, a proposed intracellular target of NO, was selectively located in presynaptic CB1R+ nerve terminals. Hence the picture was that NO was released as a retrograde signal from the PCs, affected cGMP in the presynaptic CB1R+ terminals and acted in concert with eCBs to inhibit GABA release. It was proposed that NO acted at a step downstream of CB1R, although activation of CB1R via the synthetic CB1R agonist, WIN55212-2, was not affected. It was unclear why eCB-mediated actions were immune to NO in the absence of CCh. To our knowledge, these provocative observations have not been replicated, hence although mAChRs do generate NO at neuromuscular synapses (Malomouzh et al., 2007; Newman et al., 2007), even interacting with eCBs at other synapses (e.g., via M3 activation at vertebrate neuromuscular synapses, Newman et al., 2007), a role for NO in mAChR actions in hippocampus remains conceivable but undefined.

**Figure 5:**
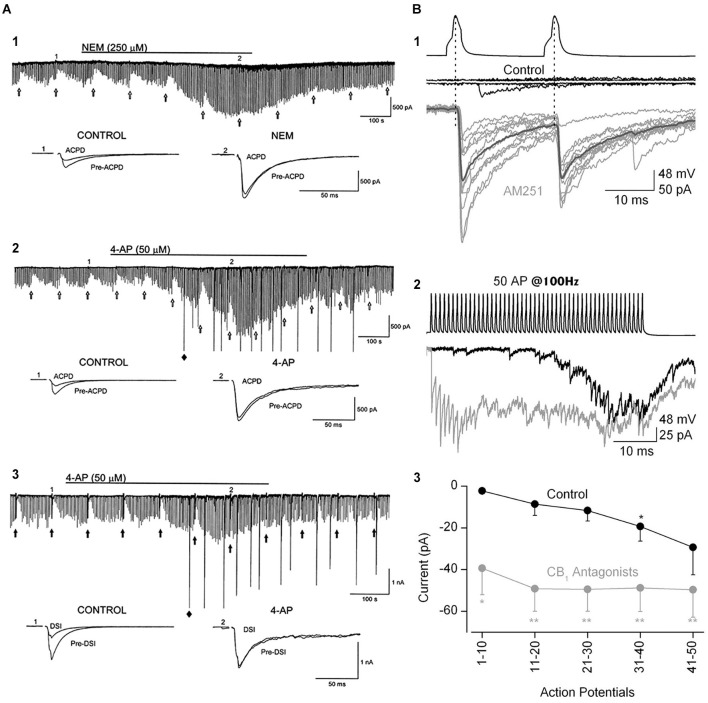
**The ability of eCBs to inhibit GABA release can be modulated by manipulations that increase transmitter release. (A1)** Bath-application N-ethylmaleimide, an organic compound that affects G-proteins, ion channels and other biochemical processes, increases GABA release and abolishes the GPCR-dependent, eCB-mediated depression of IPSCs, as well as DSI (not shown; cf. Morishita et al., 1997). **(A2,A3)** The K^+^ channel blocker, 4-AP, increases IPSCs and abolishes mGluR-dependent, eCB-mediated IPSC suppression and DSI. From Morishita et al. (1998) with permission. **(B)** Paired recording from mossy-fiber associated (MFA), CCK+ interneurons and CA3 PCs. **(B1)** Top two sets of traces show two presynaptic action potentials in the interneuron and the absence of a response in the PC in control saline. After addition of the CB1R antagonist, AM251, the interneuron action potentials reliably elicit large unitary IPSCs. **(B2)** A train of 50 interneuron action potentials initially produces only a few sparse IPSCs in the PC towards the end of the train in control solution (black trace). In the presence of AM251 (gray trace) the IPSCs are detected from the first action potential and occur throughout the train. **(B3)** Group data showing the difference in the IPSC currents, integrated within 100-ms time windows, in control and CB1R antagonist conditions. The conclusion is that a tonic, eCB mediated suppression of GABA release can be overcome by vigorous stimulation of interneuron activity. From Losonczy et al. (2004) with permission.

Resolution of the puzzle that mAChRs both mobilize eCBs and stimulate the activity of eCB-sensitive interneurons could well involve a mechanism that modulates presynaptic CB1R actions and partially offsets their depressive effects on GABA release. Several candidates exist (Figure 5), including: (1) K^+^ channel antagonists—blocking K^+^ channels pharmacologically can completely abolish DSI (Alger et al., 1996; Morishita et al., 1998; Diana and Marty, 2003), probably because voltage-gated Ca^2+^ channel opening and intra-terminal [Ca^2+^]_i_ increase when K^+^ channels are blocked (Varma et al., 2002). ACh-induced blockade of presynaptic K^+^ channels (or other factors, e.g., retrograde release of arachidonic acid; Carta et al., 2014) might thus overcome CB1R-induced depression during ACh action. (2) Direct effects on the transmitter release machinery via application of N-ethylmaleimide (NEM), which blocks pertussis toxin-sensitive G-proteins, increases GABA release and reverses DSI through an unknown mechanism (Morishita et al., 1997). (3) The firing frequency of the interneuron—the degree of eCB-mediated suppression of GABA release decreases as the firing of the interneuron increases (Losonczy et al., 2004; Földy et al., 2007). Since CCh stimulates interneuron firing (Pitler and Alger, 1992a; Martin and Alger, 1999; Cea-del Rio et al., 2010; Gulyás et al., 2010), the net effect of eCBs on persistently occurring IPSCs will represent a balance between inhibition and excitation of interneuronal output.

**Figure 6:**
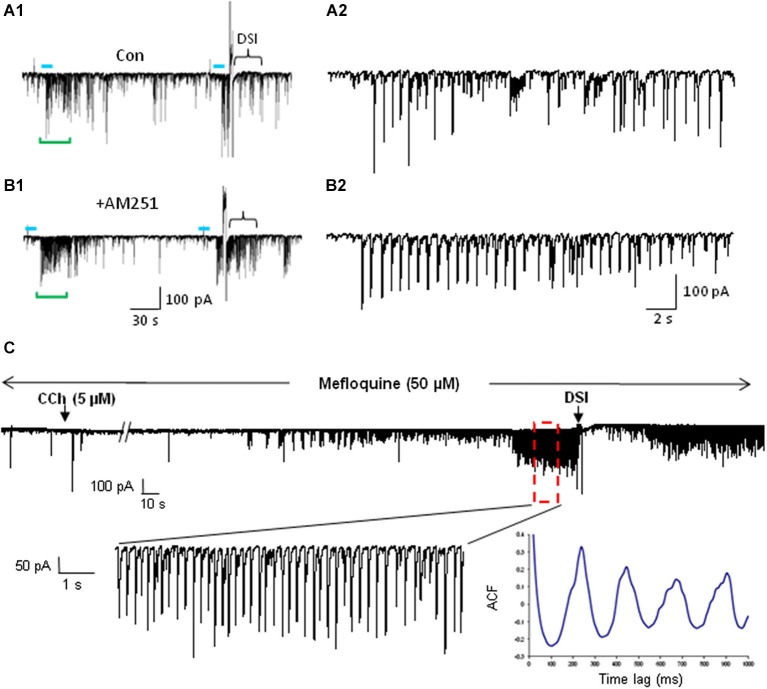
**mAChR-induced IPSCs are regulated by eCBs but do not depend on electrical coupling for their occurrence**. **(A)** Bath application of the CB1R antagonist AM251 increases the occurrence of IPSCs evoked by optogenetic release of ACh (CA1 PC recording). Blue bars indicate the period of light stimulation (5 Hz). Traces in **(A2)** and **(B2)** depict an expansion of ~29 s of the traces (green brackets) in **(A1)** and **(B1)** beginning just before the onset of L-IPSC activity. The increase in number and average amplitude of the L-IPSCs caused by AM251 indicates that they had been partially suppressed by the eCBs mobilized by ACh. Bracket at the end of **(A1)** indicates approximate period of DSI after a voltage step given to the pyramidal cell. Comparable period in **(B1)** shows that AM251 prevents DSI, thus confirming that the L-IPSC activity (e.g., expanded trace in **A2**), despite being partially suppressed by the long-lasting ACh-induced mobilization of eCBs, could be further depressed by a sudden release of eCBs (i.e., DSI). Physostigmine and 4-AP are present. **(A)** and **(B)** modified with permission from Nagode et al. (2011). **(C)** Representative trace from a rat hippocampal slice pretreated and continuously perfused with the gap junction blocker, mefloquine (50 μM). Inset shows an expanded time scale of the indicated region in the top trace. The autocorrelogram of the expanded region demonstrates rhythmic, CCh-induced IPSCs despite the presence of mefloquine. Typical results (*n* = 5). From D.A. Nagode Ph.D. thesis at http://archive.hshsl.umaryland.edu/handle/10713/2315.

If indeed mAChR-released eCBs suppress, but do not abolish, CCK+/CB1R+ interneuron output, then a CB1R antagonist should increase the IPSCs coming from these cells. That is, we predict that action of mAChRs on the interneurons will cause them to fire and release GABA, while the CB1R antagonist will prevent the eCBs generated by the PCs from simultaneously retarding the occurrence of the IPSCs. Thus a given ACh stimulus should give rise to more IPSCs in the presence of CB1R antagonism than it normally would. While concerted effort will be required to test this hypothesis in detail, we have observed (Nagode et al., 2011) that indeed the CB1R antagonist AM251 increases the number of IPSCs triggered by optogenetically released ACh (Figure 6). This example shows that the number and mean amplitude of the IPSCs triggered by ChR2-induced ACh release are increased after AM251 was applied. This suggests that eCBs generated by mAChRs can influence the IPSC rhythms. It will also be important to determine whether similar influences can be detected on atropine-sensitive, inhibitory rhythms *in vivo*, as this would suggest that mAChR-induced eCBs could be involved in regulation of persistent, behaviorally significant rhythms.

Finally, CB1R+ interneurons are electrically interconnected (Galarreta et al., 2004) and eCBs can indirectly strengthen electrical synapses (Pereda, 2014). Additionally, weakening of inhibition between electrically connected interneurons (as might occur during enhanced eCB release), also strengthens electrical coupling (Iball and Ali, 2011). Strengthening of electrical coupling will enhance the synchrony of firing within such networks. Thus complex interactions among chemical and electrical synapses and eCBs could help to rationalize the role of mAChRs in θ inhibitory oscillations. Unraveling the details of the modulation of eCB actions initiated by the cholinergic system will be an important task for the future.

## mAChR driven eCB release and electrical synaptic connections sharpen distinctions among interneuron circuits and tune interneuronal oscillations

The two major BC interneuron subtypes, the CCK+ (regular-spiking, RS) and the PV+ (fast-spiking, FS) cells are sharply segregated by their divergent properties (Freund and Katona, 2007), including their complements of mAChRs. These cellular and molecular differences imply that the two cell types are activated under different circumstances, by different neurotransmitters and modulators, and cause different effects on their target cells. Another factor is critical to ensuring that the cells within each group do not act in isolation, but participate in coordinated circuit based activity. As noted, electrical synaptic coupling often exists among like cells in hippocampus and neocortex (Galarreta and Hestrin, 1999; Gibson et al., 1999), although some interneurons are electrically coupled to cells of different classes (e.g., Krook-Magnuson et al., 2011). Generally, PV+ cells are electrically coupled to other PV+ cells, but not to PCs or other types of interneurons, including the CCK+ cells. CCK+ cells are electrically coupled to other CCK+ cells (Galarreta et al., 2004), but not to PCs or other types of interneurons, including the PV+ cells. The steady-state electrotonic coupling (coupling coefficient) among the cells averages from 4–7%, meaning that, e.g., a 10 mV change in membrane potential in one cell changes the potential in the coupled cell from 0.4–0.7 mV. This is enough to induce a cell to fire if it is near threshold. Injection of two random noise signals (Galarreta and Hestrin, 1999) or small sinusoidal voltages (Gibson et al., 1999) into two electrically coupled interneurons can cause both of the cells to fire synchronously when either of them reaches threshold. Thus the PV+ cells would tend to fire together as a circuit and the CCK+ cells would also tend to fire as an independent circuit. Interneuron circuit-wide activity will powerfully influence PC population activity.

The existence of electrical gap junctions between the cells is also noteworthy because it confers susceptibility to modulation by various regulatory factors. The strength of gap junctional transmission is dependent on the input (leak) resistance of the target cells, and is frequency dependent, decreasing as the frequency of the voltage deflections through the junctional channels increases. When the leak resistance is low the coupling among cells is also low, because the currents, instead of passing via the gap junction into the coupled cells and depolarizing them, are shunted through the leak resistance to the extracellular space. When the leak resistance is high, the strength of electrical coupling increases. Interestingly the PV+ and CCK+ interneurons that are electrically coupled to each other are frequently also chemically coupled; that is, the target postsynaptic cells receive both electrical and chemical synaptic transmission from their upstream presynaptic partners (Galarreta and Hestrin, 1999; Gibson et al., 1999; Ali, 2007; Iball and Ali, 2011). The release of GABA from CCK+ cells can be suppressed by eCBs (Ohno-Shosaku et al., 2001; Wilson and Nicoll, 2001), and when eCBs are released from other CCK+ cells the eCBs reduce the strength of chemical inhibition (Iball and Ali, 2011). By decreasing the chemical synaptic inhibition among CCK+ cells this simultaneously strengthens the electrical coupling between them. The release of eCBs depresses the strengths of individual GABAergic synapses onto other CCKs cells *and* increases their tendency to fire together. Importantly, the two kinds of synaptic junctions are independently regulated in the hippocampus; eCBs only suppress the release of GABA, they do not affect the electrical coupling. Thus inhibition can do more than simply veto or permit cell firing, it can directly shift the mode of firing within interneuronal circuits. The functional aspects of this concept has not been explored in the context of mAChR control of oscillations. However, given the ability of mAChRs to mobilize eCBs, mAChR-dependent stimulation of CCK+ cell mediated oscillations could in part reflect dual regulation of chemical and electrical signaling, although the gap junction blocker, mefloquine (Cruikshank et al., 2004), did not alter the ACh-induced θ IPSCs (Figure 6C).

PV+ cells are also electrically as well as chemically coupled and their tendency to fire together is facilitated by electrical synapses (Galarreta and Hestrin, 1999). Activation of mAChRs induces the occurrence of γ rhythms driven by PV+ cells in hippocampal CA3, but PV+ cells but do not express CB1Rs and are therefore not directly affected by eCBs. The PV+ cell-mediated inhibitory γ rhythms are suppressed by *exogenous* cannabinoids because activation of the CB1Rs on the glutamate terminals that excite the PV+ cells is suppressed and the resulting loss of excitatory drive keeps the cells from firing (Holderith et al., 2011). Surprisingly, the eCBs released by activation of M1/M3 mAChRs on PC apparently do not affect the CB1Rs on the glutamate terminals. This conclusion follows from the observation that the CB1R antagonist that prevents the inhibition of γ by exogenous eCBs, when applied by itself does not alter mAChR-driven oscillations (Gulyás et al., 2010). Given that a mAChR agonist very efficiently suppressed GABA release via eCB action in these experiments, it is clear that the eCBs were mobilized. Probably the eCBs simply did not reach the CB1Rs on glutamatergic terminals. The powerful eCB uptake and degradation systems, together with the fact that eCBs cannot travel far from their site of production/release (Kano et al., 2009) could have limited their movements. The restricted actions of the eCB system help sharpen the targeting of ACh actions, even if ACh is released in the volume conduction mode.

Release of GABA from PV+ cells is regulated by opioids, because these cells strongly express μORs on their nerve terminals (Drake and Milner, 2002). Thus μOR agonists, such as enkephalins, may have the analogous effects on the development of PV+ driven inhibitory rhythms as eCBs do on the CCK+ cell rhythms. Mobilization of endogenous opioids in the hippocampus by ACh has not yet been explored in this context to the best of our knowledge.

**Figure 7:**
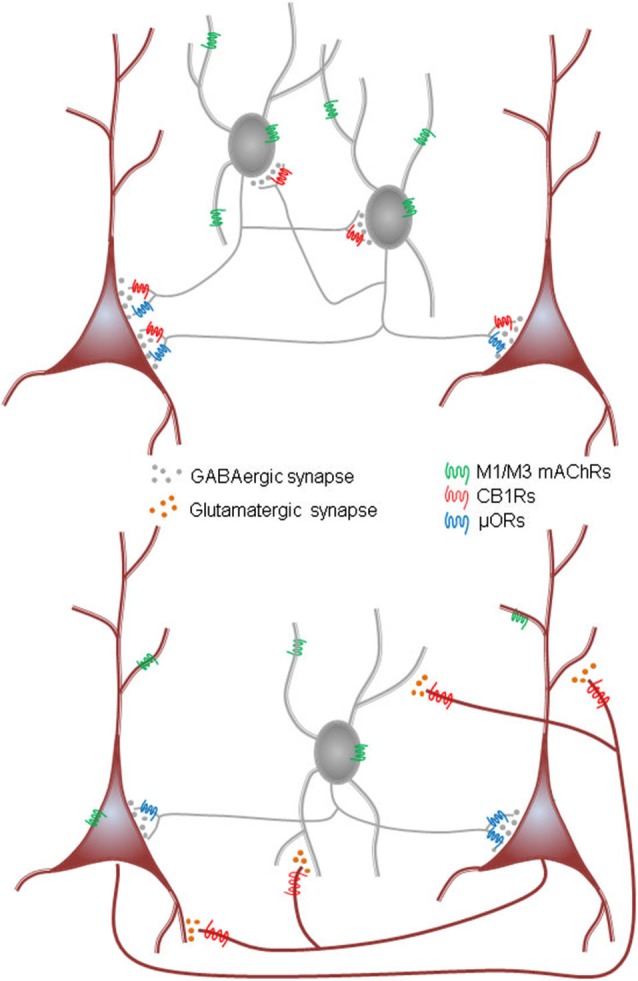
**Diagrams of two models for mACh-induced inhibitory rhythmic IPSCs in hippocampus**. **Top**, synaptically connected interneuron network is tonically activated by activation of M1/M3 mAChRs on interneurons in CA1. Interneuron firing is induced by mAChR-induced depolarization that, when integrated with intrinsic interneuron firing properties and incoming GABAergic IPSPs from other interneurons of the group, generates rhythmic synchronous interneuron firing. The target PCs receive a rhythmic barrage of IPSPs. This is analogous to the ING (“interneuron gamma”) model of gamma rhythms. Cannabinoids interrupt rhythms generated by this network by inhibiting the release of GABA from the CB1R-expressing (mainly CCK+) interneurons; opioids probably inhibit the network by acting on μORs present on a subset of the CB1R+ cells. Note evidence of Pietersen et al. (2014) for an intrinsic γ generator in CA1 that would involve a PING mechanism. **Bottom**, ACh drives action potential firing in an interconnected excitatory network (such as the CA3, but not the CA1, PCs) as well as in the interneurons. The glutamatergic output of the PCs excites interneurons that feed GABAergic IPSPs back onto the PCs. Interactions between the excitatory and inhibitory cells generates the rhythms. This is analogous to the PING (“pyramidal-interneuron gamma”) model. Cannabinoids inhibit rhythms generated by this network by inhibiting the release of glutamate from the PCs; opioids inhibit the rhythms by acting on the μORs on the (mainly PV+) interneurons.

It is established that the CCK+ cells and the PV+ cells predominate in different kinds of neuronal oscillations. The preferred frequencies of the oscillations, γ for the PV/FS cells and the slower θ rhythms for the CCK/RS, cells will largely be set by intrinsic membrane properties, including the kinetics of their AHPs, that enable the PV/FS cells to fire at higher frequency than the CCK/RS cells, as well as by the kinetics of the chemical transmission that they each mediate—IPSPs mediated by PV/FS cells are faster than those of the CCK/RS cells. Most importantly, the circuitry underlying the rhythms, at least in the hippocampus, is likely to be quite different. The inhibitory θ in CA1 is probably generated by an interconnected inhibitory network of CCK+ cells that express CB1Rs, and perhaps also μORs. This rhythm is independent of fast excitatory glutamatergic synaptic input. Rather, the rhythmic output of this circuit is produced when the cells receive a slow cholinergic input that activates their mAChRs for at least several seconds. Inhibitory synaptic interactions among the interneurons then gives rise to synchronous rhythmic firing within the network, and IPSCs are projected onto groups of PCs. In contrast, the faster inhibitory γ rhythms in CA3 are generated mainly by excitatory synaptic interactions among CA3 PCs, which activate PV+ interneurons that then feed back inhibitory inputs to the PCs. These rhythms are abolished by iGluR blockers, or CB1R agonists, which prevent stimulation of the PV+ cells; they are also abolished by activation of μORs on the PV+ cell terminals. The schematic diagram in Figure 7 summarizes these conclusions. Note that this schematic is intended only to illustrate the circuitry for the inhibitory rhythms, it does not include other circuitry such as that described by Pietersen et al. (2014) that produces an intrinsic γ rhythm that is entrained by cholinergic inputs and is dependent on excitatory synaptic inputs (hence a PING model) in CA1.

### Future areas for exploration of mAChR function in the brain

Despite the enormous amount of investigation into muscarinic cholinergic systems in the brain there are still many areas about which little is known. We highlight a few opportunities related to their roles of in hippocampal and cortical oscillations or behaviors related to them.

#### mAChRs, PAMs, and endocannabinoids

Deficiencies in mAChR actions are implicated in various kinds of cognitive dysfunction. Attempts to develop effective therapeutic agents that act directly on specific brain mAChRs have not been successful, largely because of difficulties in restricting the agents to particular mAChR subtypes (Bubser et al., 2012). These agents are generally agonists that bind to the active site of the molecule, or generally enhance the availability of ACh (by preventing its uptake, for example). In either case, side effects occur when unintended receptors are also activated. It has been difficult to devise agonists that only activate one mAChR subtype because the agonist binding site is highly conserved across subtypes. An alternative approach targets sites that are away from this highly conserved region. Ligands at these sites, allosteric modulators, are more specific because they bind to relatively less well-conserved parts of the receptor molecule, i.e., sites that vary widely between subtypes and hence offer more opportunities for specific binding. Allosteric modulators do not directly activate the receptor but enhance the effects of ACh or other ligands that activate it directly. For example, positive allosteric modulators (PAMs) that are specific for M1, M4 or M5 mAChRs have been developed (Bubser et al., 2012). In the presence of a PAM for M1, a low concentration of ACh that produces predominantly M1 mAChR dependent effects would increase the ability of naturally released ACh to activate M1 selectively on its normal postsynaptic target cells. This approach should improve the specificity of action over the usual systemic therapeutic drug application method. Similar strategies have been used in the case of nAChRs; e.g., the weak AChE inhibitor and PAM of nAChRs, galantamine, attenuates nicotine self-administration and seeking rats (Hopkins et al., 2012).

Because activation of M1 (or M3) mAChRs potently stimulates the release of eCBs, an M1-specific PAM should enhance the ability of cholinergic agonists to mobilize eCBs. This hypothesis has not been tested, but could easily be investigated in *in vitro* brain slice preparations. If PAMs do facilitate eCB mobilization by mAChRs, it might have clinical applications, particularly in view of the preliminary results suggesting efficacy of CB1R agonists in alleviating certain consequences of Alzheimer’s dementia (agitation, lack of nutritional intact, sleep disturbances; Aso and Ferrer, 2014). Perhaps a mAChR PAM given in conjunction with low concentrations of a CB1R agonist would be beneficial and further reduce the possibility of untoward side effects of either drug alone. Alternatively, an eCB uptake inhibitor, by increasing the concentration of eCBs near their normal site of action, might be beneficial in boosting the eCB mobilizing ability of M1 mAChR activation.

#### mAChRs, glia, and eCBs

No longer thought to be passive supporting partners of neurons, glia are now understood to have active roles in the regulation of synaptic transmission. Glial cells, mainly astrocytes, express a diversity of mAChRs including M1 and M3 (Pap et al., 2009). In several brain regions activation of mAChRs on glia cause elevations in intracellular glial [Ca^2+^]_i_ (Araque et al., 2002; Pap et al., 2009; Takata et al., 2011; Navarrete et al., 2012). Glia participate in the induction of synaptic plasticity in the hippocampus, including a form of LTP at the CA3-CA1 synapses that is dependent on activation of mAChRs (Navarrete et al., 2012). Stimulation of glial mAChRs by application of cholinergic agonists, or stimulation of ACh release from septal cholinergic fibers causes an increase in hippocampal Ca^2+^ in glia (Araque et al., 2002). *In vivo* sensory stimulation or electrical stimulation of the MS increases Ca^2+^ in hippocampal astrocytes and induces LTP of CA3-CA1 synapses (Navarrete et al., 2012). This cholinergic LTP induction depends on activation of mAChRs and mGluRs. Rises in glial cell Ca^2+^ result from activation of IP_3_Rs (Takata et al., 2011; Navarrete et al., 2012), and are associated with the release of various factors (Sul et al., 2004) including glutamate (Halassa and Haydon, 2010, for review). Astrocytes are also activated by endocannabinoids (Navarrete and Araque, 2008; Min and Nevian, 2012). The glial induction of LTP in the hippocampus is caused by Ca^2+^-dependent glutamate release from the astrocytes and subsequent activation of hippocampal PC mGluRs. A similar cholinergically-driven, astrocyte-Ca^2+^ mediated synaptic plasticity in the mouse barrel cortex is dependent on mAChRs and NMDARs (Takata et al., 2011), indicating that mAChR activation stimulates glutamate release there as well. Given that glutamate activation of mGluRs is a potent stimulus for eCB mobilization from PCs (Maejima et al., 2001; Varma et al., 2001) elevation of glial cell [Ca^2+^]_i_ should also mobilize eCBs indirectly from the PCs following mGluR stimulation. Given the eCB-mediated influences on cortical and hippocampal rhythms, glia could also participate in regulation of rhythms via eCBs. This hypothesis has evidently not been tested, but if true, would add another potent element to the array of effects mediated by mAChR.

#### mAChR regulation of rhythms by controlling ectopic axonal activity

It is generally assumed that axons simply transmit signals from neuronal somata to synaptic terminals, and therefore that they automatically follow somatic activity. Two corollaries follow from this assumption: (1) somatic action potential activity is an accurate guide to the activity reaching the terminals; and (2) axons do not act independently of somata.

However, under some circumstances axonal action potentials can be initiated independently of somatic depolarizations. These were initially described in the context of disease or other aberrant conditions, but new challenges to the simple picture have arisen in physiological contexts. In hippocampal slices, Dugladze et al. (2012) report that kainic acid-induced γ oscillations in the field potentials around the PCs of the CA3 region are accompanied by much higher frequency firing in the distal axonal branches of the PCs. Remarkably, this high frequency of axonal firing was not reflected in the somatic action potential firing of the PC somato-dendritic regions. It appeared that the two cellular compartments—axon and soma-dendrite—were in essence operating independently. When GABA-A receptors were pharmacologically blocked, however, the axonal action potentials did invade the somato-dendritic region, implying that normally they were actively prevented from doing so by a persistent GABAergic inhibition. The investigators discovered that a continual high frequency firing of the axo-axonic interneurons, which specifically target the axon hillock region of the PCs, were responsible. In fact, a single axo-axonic cell was capable of fully controlling the antidromic invasion of the somato-dendritic region of a PC. As noted earlier, the axo-axonic cells in CA3 are strongly activated by mAChR activation, but the IPSPs produced by these cells do not directly contribute to oscillations. Rather, Dugladze et al. (2012) suggest that the main function of the axo-axonic cells is to preserve the independence of axonal and somato-dendritic signaling. It will be important to determine if mAChR-induced oscillations share the ability to modulate PC function in this novel and powerful way.

#### mAChRs, eCBs, and Fragile X Syndrome

Endocannabinoid modulation of CCK+ cells may underlie some of the deficits in oscillations in Fragile X Syndrome (FXS). In the hippocampus of a mouse model of FXS, *Fmr1^−/–^* mice, there is enhanced coupling of mGluRs to eCB release at inhibitory synapses in both hippocampus (Zhang and Alger, 2010) and striatum (Maccarrone et al., 2010). Surprisingly, the eCB actions at excitatory synapses are actually decreased, not increased, in this model (Jung et al., 2012). While the molecular basis for this striking difference is not fully understood, the distinctive molecular architecture of excitatory and inhibitory synapses will undoubtedly constitute a major factor. DGL_α_ is normally precisely localized in the spine heads of excitatory synapses (Katona et al., 2006), and it has been found that the disease is associated with an enhanced distance between mGluRs and DGL_α_ (Jung et al., 2012) which could explain the decreased efficiency of eCB production. DGL_α_ has not yet been found at inhibitory synapses (e.g., Lafourcade et al., 2007), and the explanation for enhanced eCB actions at those synapses is unknown, although biochemical targets are being identified (Busquets-Garcia et al., 2013). It should be emphasized that the functional consequences of both decreased eCB action at excitatory synapses and increased eCB action at inhibitory synapses will be the same: an overall increase in network excitability. The same could be true of mAChR-induced eCB release, as overactive signaling through M1 mAChRs has been hypothesized to contribute to the FXS phenotype (D’Antuono et al., 2003). There is enhanced CCh-induced LTD in CA1 hippocampal slices from FRX mice (Volk et al., 2007), and M1 and M4 antagonists reduce the induction of audiogenic seizures (Veeraragavan et al., 2011a,b). The relationship between mAChRs and eCBs deserves further study in the context of FXS, in part because the availability of clinically tested CB1R ligands that could be candidates for inclusion in the therapeutic arsenal for treatment of symptoms of this serious disorder.

## Conclusion

Studies of the mAChR system in the brain continue to yield exciting new insights and information on a wide variety of neurophysiological problems. Undoubtedly the future holds enormous promise for novel and valuable advances both in the basic understanding of this powerful and ubiquitous regulatory system, and in eventual clinical applications.

## Contributions of authors

This review was written jointly by all of the authors. The recent research referred to from the Alger laboratory was supervised by Bradley E. Alger and conducted by Daniel A. Nagode and Ai-Hui Tang, who also had major roles in the experimental design, data analysis, and interpretation of results.

## Conflict of interest statement

The authors declare that the research was conducted in the absence of any commercial or financial relationships that could be construed as a potential conflict of interest.
